# A brief review of *in vitro* models of diabetic neuropathy

**DOI:** 10.4103/0973-3930.57344

**Published:** 2009

**Authors:** Namita G. Hattangady, Medha S. Rajadhyaksha

**Affiliations:** Department of Life Sciences, Sophia College, B. Desai Road, Mumbai - 400 026, India

**Keywords:** Diabetic neuropathy, high glucose, *in vitro* experimental models

## Abstract

The neuropathies of the peripheral, central and autonomic nervous systems are known to be caused by hyperglycemia, a consequence of the deregulation of glucose in diabetes. Several *in vivo* models such as streptozotocin-induced diabetic rats, mice and Chinese hamsters have been used to study the pathogenesis of diabetic neuropathy because of their resemblance to human pathology. However, these in vivo models have met with strong ethical oppositions. Further, the system complexity has inherent limitations of inconvenience of analyzing ephemeral molecular events and crosstalk of signal transduction pathways. Alternative *in vitro* models have been selected and put to effective use in diabetic studies. We critically review the use of these in vitro models such as primary cultures of dorsal root ganglia, Schwann cells and neural tissue as well as neural cell lines which have proved to be excellent systems for detailed study. We also assess the use of embryo cultures for the study of hyperglycemic effects on development, especially of the nervous system. These systems function as useful models to scrutinize the molecular events underlying hyperglycemia-induced stress in neuronal systems and have been very effectively used for the same. This comprehensive overview of advantages and disadvantages of in vitro systems that are currently in use will be of interest especially for comparative assessment of results and for appropriate choice of models for experiments in diabetic neuropathy.

## Introduction

Diabetic neuropathy is a painful complication involving progressive neuronal damage and dysfunction. It affects the sensory nerves,[1] the autonomic nervous system[2,3] and even the central nervous system (CNS).[4] Increasing evidence indicates that one of the major causes of diabetic peripheral neuropathy is an over-production of reactive oxygen species which leads to oxidative stress,[5–8] mitochondrial dysfunction,[9,10] neuronal damage[11] and finally apoptosis.[12,13] The effect of diabetes on the central nervous system has also been recorded in the form of changes in the structure of the blood brain barrier,[4] neurophysiology,[14] increased neuronal apoptosis[15–17] and reduction in cognitive abilities.[18,19] On the other hand, evidence challenging neuronal death by apoptosis and survival mechanisms in operations has also been reported.[20–23] This information has accumulated by using a variety of *in vitro* and *in vivo* experimental models.

Several animal models of diabetic neuropathy have been developed which include streptozotocin-induced (STZ-induced) diabetic rats,[24,25] STZ-induced diabetic mice,[26–27] Chinese hamsters[28] and Otsuka Long-Evans Tokushima Fatty (OLETF) rats.[29,30] Each has been used appropriately to address specific questions because of their part pathological resemblance to humans, their amenability to genetic manipulation and/or generation of transgenic animals.[31,32] While *in vivo* studies provide physiological and cellular details of diabetic neuropathy, the system complexity present obscures the details of fleeting molecular events that may decide cell death or survival under glucose stress. The use of simpler *in vitro* systems is hence imperative to circumvent these problems.[10,12,13] Despite strong ethical justification, very few such *in vitro* models have been developed. The most extensively used *in vitro* models are primary culture of dorsal root ganglia neurons and the neuroblastoma cell line. Here, we critically review the advantages and disadvantages of *in vitro* systems used as models of diabetic neuropathy. Table 1 summarizes such *in vitro* models and their effectiveness in the study of diabetic neuropathy.

**Table 1:** *In vitro* models used for studies on diabetic neuropathy

	Major findings	Models	References
Category	Details of events		
Mitochondrial events	Change in membrane potential, change in morphology and number, changes in enzymes and membrane proteins	DRG, Schwann Cells, SHSY5Y	[16,35,16,53]
		DRG, Schwann Cells, HSY5Y	[10,16,53]
		DRG, cortical neurons, HSY5Y, PC12, rodent	[10,13,41,53,57,58,66]
Apoptotic changes	Caspase activation, other apoptotic changes	DRG, Schwann Cells, cortical neurons, SHSY5Y, PC12	[13,15,34,35,16,41,16,53,59]
		DRG, Schwann Cells, cortical neurons, PC12	[10,13,15,34,35,36,43,41,57,58,59]
Generation of ROS		DRG, SHSY5Y, PC12, rodent	[13,34,35,6,54,59,60,66]
Other cellular and enzymatic changes		DRG, Schwann cells, cortical neurons, neural crest cells, SHSY5Y, schwannoma, PC 12, rodent	[36], [43]
			[41,45],[54],[62,61,58,59,60,64,66]
Neuroprotection studies		DRG, Schwann cells, cortical neurons, neural crest cells, SHSY5Y, schwannoma, PC 12	[13,15,16,34,35,36],[16,41]
			[45,16,53] [60,66]
Regenerative studies/no apoptosis		DRG/ Schwann Cells	[20,21,22]

## Primary Cultures as a model system

### Dorsal root ganglia neurons

Neurons isolated from dorsal root ganglia (DRG) of mammals such as rodents and hamsters rendered diabetic by treatment with drugs such as streptozotocin and alloxan[16,24,33] have been used for study of diabetic neuropathy. Use of this system has its share of controversies arising because of delays in collection of sample and possible loss of critical time points, thus providing little scope to analyze spatial and temporal details of molecular events occurring in neuronal dysfunction. Further, rendering an animal diabetic is met with strong and legitimate ethical opposition. Primary culture of DRG neurons from normal untreated mammalian embryos are now preferred as the next best candidate[10,13,16,33,34] as they partly overcome ethical opposition, mimic events occurring *in vivo* to some extent and permit detailed molecular analysis.[35–37] It is important to note that some biological attributes such as responsiveness to growth factors vary in DRG neurons of embryonic and adult origin.[38]

Primary cultures of DRG neurons harvested from normal animal systems has been cultured in an appropriate growth media,[10,13,17,34,36] thus overcoming the interference of hormones and external agents that may modulate behavior of cells in hyperglycemic conditions. Further, conspicuous changes in morphology such as reduction in neurite extension,[39] changes in the activity of enzymes involved in the tricarboxylic acid cycle, electron transport chain, antioxidant systems[19] and molecular events involved in mitochondrial dysfunction leading to apoptosis.[9,10,16,37] have been demonstrated using primary cultures of DRG neurons.

DRG primary cultures are also convenient systems to trace the time kinetics of the molecular events occurring during cell death due to oxidative stress.[13,16,34] Most of these events have been shown to peak between 1-3 h after treatment with glucose due to sudden increase in ROS and associated stress.[13,16,34] These observations concur with *in vivo* studies.[37] A study by Vincent *et al*, proposes that DRG neurons are able to adapt through feedback mechanisms, by reducing aconitase and by increasing their antioxidant enzyme activity, thereby preventing additional stress in case of a second glucose insult. Thus, DRG cultures have been effectively used to study feedback mechanisms and cumulative molecular effects in acute hyperglycemia.[13] They are useful in checking the efficacy of neuroprotectants such as uncouplers.[16,35]

In an excellent study by Gumy *et al*, culture of ganglia of adult mouse was established to quantify apoptosis as well as regenerative response of DRG neurons and Schwann cells. Interestingly, neuronal apoptosis was found to be negligible while cell migration and proliferation of Schwann cells was impaired. These observations challenged the notion that hyperglycemia leads to neuronal apoptosis.[20] This was supported by another study where sensory neurons from STZ-treated rats, though expressed heightened Caspase 3 activity and peroxynitrite toxicity there was no evidence of apoptosis as monitored by transferase mediated dUTP nick-end labeling (TUNEL) technique. On the other hand, there was evidence of repair cascade in operation as seen by upregulation of DNA repair enzyme poly (ADP-ribose) polymerase (PARP).[22] Support for hyperglycemia-induced regeneration has come from explant cultures of DRG as well.[23]

Despite the merits, the use of DRG primary cultures has some serious limitations, one of them being the presence of contaminating neuronal cells such as fibroblasts and Schwann cells.[35] Although the growth of non-neuronal cells can be reduced by treatment with 5′-fluoru-2′deoxyuridene[34,36,40] or cytosine-D-arabinofuranoside,[41] this is an important concern as co-cultures can yield results which are markedly different from monocultures. In fact, Vincent *et al*, report that, under similar hyperglycemic conditions, cultured embryonic rat DRG neurons that were depleted of Schwann cells showed more susceptibility to caspase activation than DRG neurons co-cultured with Schwann cells or mature rat DRG neurons cultured with satellite-like supporting cells.[35] On the other hand, this ability of DRG neurons to grow as co-cultures can be used as an advantage for addressing questions regarding the specific roles of surrounding cells in normal physiology.

### Schwann cells

Primary cultures of Schwann cells from Sprague-Dawley rat embryos have been exposed to high glucose *in vitro* to investigate the pathogenesis of diabetic neuropathy.[16] Such studies report hyperglycemia-induced molecular events which concur with those reported in DRG neurons.[37] However, isolating maximum possible Schwann cells from a fresh nerve is difficult because the abundant connective tissue cells and other myelinating cells often overwhelm Schwann cells.[42] They do not readily attach to a substratum and show poor proliferation, many cells dying within 24 h of plating.[42] Further, Schwann cell cultures have had their share of controversies regarding their ability to mimic certain *in vivo* events such as a change in the activity of aldose reductase.[33,43]

### Neural crest cells

Neural tube fusion demonstrated in Sprague-Dawley rats treated with STZ suggests teratogenic effects of hyperglycemia on the developing nervous system.[44] An *in vitro* model was developed by Suzuki *et al*, using primary culture of neural crest cells of embryos from the malformation prone substrain of STZ-induced diabetic female rats on gestation day nine. This study demonstrated that exposure to high glucose reduces cell number and inhibits cell migration.[45] This system was also used efficiently to demonstrate the neuroprotective effects of antioxidant N-acetylcysteine and superoxide dismutase against hyperglycemia.[45]

### Cortical neurons

Neuropathy of the CNS in various *in vivo* models as well as in human diabetic patients has been extensively reported in the form of cognitive deficits.[4,18,19] To determine the cellular and molecular events underlying these defects, *in vitro* short term cultures of cortical neurons from diabetic rats have been developed and used to demonstrate an increase in apoptosis and decrease in neurite formation as a consequence of hyperglycemia.[41] Molecular events involved in retinoic acid signaling indicate that exogenously administered retinoic acid can help avoid CNS complications *in vivo.*[41] *In vitro* systems thus provide data which can be used to develop therapeutic strategies against hyperglycemia-induced CNS neuropathy.

## Organotypic Cultures

Developed by Hogue in 1940, organotypic cultures involve the culturing of slices of the brain in monolayers in rolling tubes or coverslips.[46] This technique has an edge over other *in vitro* systems in that the tissue retains its original connective and structural organization thus mimicking exact physiological conditions.[46] Slice cultures of DRG neurons have been reportedly maintained for almost ten days and can be used for electrophysiological studies.[46] After undergoing several modifications, cover slips have now been replaced by culture plates coated with adherent protein.[47] Such cultures have already proven to be efficient tools in investigating ischemic neurodegeneration in the hippocampus and in testing neuroprotective drugs.[48–50] An elegant study has been carried out using explant cultures of DRG and nodose ganglia by Sango *et al*, which demonstrates that in contrast to other reports, high glucose was not a potent killer of neurons but may cause regenerative changes seen as neuritogenesis.[23] The use of organotypic cultures has substantially resolved the ethical issues of using STZ-induced diabetic animals for studying electrophysiological parameters, axonal transport and changes in axon morphology. Reports of impairment in slow transport and changed morphology of axons in STZ-induced diabetic mice[51,52] have emphasized the need to create efficient models to study such parameters.

In conclusion, primary cultures of neural cells have provided valuable data on high glucose-induced cellular damage. However, these systems are relatively heterogeneous due to the presence of rapidly dividing, non-neuronal contaminating cells such as fibroblasts.[47] Moreover, cells undergo alteration in their properties, cease to divide and eventually die out, surviving only a few passages *in vitro.*[46] This limits the use of techniques that require large cell numbers.[47] To overcome some of these drawbacks, transformed cell lines came into use.

## Transformed Cell Lines as a Model System

A variety of cell lines have been used to study diabetic neuropathy, depending on the questions being addressed. Most of these cell lines are easily obtainable and can be cultured to form infinite passages.[47] Cells also extend processes to form networks and become electrically active.[46] They can synthesize neurotransmitters and express neurotransmitter receptors, thus resembling human physiological state.[46] These cell lines are now used as robust models to unravel the molecular basis of glucose induced changes.[53–61]

### Neuroblastoma cell line

One of the most extensively used cell lines to study diabetic neuropathy is the neuroblastoma cell line SHSY5Y,[53,54] a thrice cloned sub-line of the neuroblastoma cell line SK-N-SH established in 1970 from a metastatic bone tumor.[55] These cells, when treated with high glucose, show results similar to those observed using DRG neurons and Schwann cells.[16] In addition, SHSY5Y cells have also been used effectively to demonstrate changes in signal transduction.[54] Pathways that lead to abnormal nitric oxide production and changes in the activity of the Na^+^/K^+^ Pump have also been studied.[54] Neuroblastoma cells have also been extensively used to screen the efficacy of uncouplers[16] and insulin-like growth factor-1[53] as neuroprotectants and to investigate their mechanism of action.

### Pheochromocytoma cell line

The pheochromocytoma (PC12) cell line, derived from catecholamine secreting adrenal chromaffin tumor cells in rats,[56] has been recognized as a robust model to study neuronal dysfunction in elevated glucose levels.[56–60] The cell line has been used successfully to investigate the involvement of pro-apoptotic mitochondrial protein Bax,[57] occurrence of nuclear fragmentation,[57] carbonyl-induced stress,[58] reactive oxygen species,[59] nerve growth factor signaling[59] and nitric oxide-induced stress.[60] Neuroprotection studies have also been carried out *in vitro* using tetrahydrobiopterin, a cofactor for nitric oxide synthase.[60] The level of differentiation of PC12 cells, however, needs to be taken into account during analysis of results as naive and differentiated cells may yield entirely contradictory results. This contradiction has been reported in a study by Sharifi *et al*, where it was observed that the same elevated glucose level required seven days to reduce viability in naïve PC12 cells[57] as against three days in differentiated PC12 cells in a study by Koshimura *et al*.[60]

### Schwann cell line

Effect of hyperglycemia has been studied on immortalized cell lines of Schwann cells of various origins.[61,62] Cell line derived from adult mouse, IMS32, which is positive for S100 and p75^NTR^ immunoreactivity, expression of laminin, essential transcription and neurotrophic factors, has been efficiently used in detailed studies on diabetic neuropathy by Sango *et al*.[62] This study reports that exposure to elevated glucose leads to events such as intracellular sorbitol accumulation, enhanced activity of enzyme aldose reductase and upregulation of certain essential genes.[62] Schwannoma cell line JS-1 derived from rat has been used to demonstrate significant decrease in expression of PKC-α and decrease in proliferation.[61] An advantage of cell lines is that unlike primary Schwann cell cultures, they closely mimic *in vivo* events such as hyperactivity of polyol pathway.[61,62] However, there are limitations also, for example the IMS32 cells form abnormal ball-shaped colonies in confluent cultures and also fail to myelinate mouse axons as do primary Schwann cell cultures.[62]

In general, while cell lines are excellent models for the study of diabetic neuropathy, some inherent problems need to be addressed. For example, clonal cells are incapable of developing dendrites, axons or forming myelin.[46] Further, the uniform extent of exposure of cells to high glucose in monocultures fails to mimic *in vivo* heterogeneous conditions where cells are exposed to variable glucose levels. In fact, Halliwell proposes that cells in culture do not represent behavior of cells *in vivo* because the cell culture media tends to catalyze the oxidation of compounds added to the media. More ROS are produced in culture since reactions occur at almost 95% oxygen (O_2_), 5% carbon dioxide (CO_2_) and 150 mm HgO_2_ conditions.[63] These conditions are limited in physiological environment where cells are exposed to lower oxygen concentrations (1-10mm Hg). Finally, the presence of metals such as iron in different forms in media and calf serum also exacerbates the effect of high glucose.[63] However, it may be argued that as the protective effects of intercellular physiological antioxidants in environment are minimal, the observation of the role of intracellular antioxidant responses to hyperglycemic stress becomes easily discernable. Another hindrance to the use of cell culture models is the possibility that the effect of glucose may be due to osmotic shock. However, this has been dealt with by the use of controls treated with mannitol[15,43] or fructose.[55] Hence, results from *in vitro* studies can be extrapolated to *in vivo* situations, but with considerable skepticism.

## *In vitro* Models of Whole Embryo Culture

### Rodent whole embryo culture

The rodent whole embryo culture is a comparatively old technique devised by Dr. New[46,64,65] and consists of the culture of embryos in the head-fold stage from an euthanized diabetic pregnant animal in rotating bottles containing media and the serum of diabetic animals.[46,64] This technique is thus ideal to determine the teratogenesis induced by hyperglycemia in the embryos of type-1 diabetic mothers[64,65] and can be modified depending on the requirement of the experiment.

The rodent whole embryo culture has also been used successfully for detailed molecular studies. A study by Akazawa reports that exposure to a high glucose condition downregulates the expression of glucose transporter GLUT-1 after 48 h of glucose treatment.[66] An increase in ROS formation, deficiency in *myo-*inositol, accumulation of sorbitol, increase in the activity of the hexamine pathway and an increases the number of apoptotic cells which show upregulation of Bax protein and activation of caspase 3 has been demonstrated using this system.[66]

### Shell-less cultures of chick embryos

In an interesting study by Datar and Bhonde using the chick embryo by the shell-less culture technique. Whole embryos along with the associated yolk and thin albumin were cultured in a sterile bowl at 37°C in 80-85% humidified atmosphere.[67] The study demonstrated increased mortality and exencephaly in embryos after treatment with high glucose for 24 and 48 h.[67] This technique serves as an excellent model to study hyperglycemia-induced embryopathies including abnormalities of the developing nervous system. Since interferences due to other conditions like hypoinsulinemia and ketoacidosis can be eliminated and osmotic changes accounted for, this technique also allows narrowing down to a single causal factor.[67] Datar and Bhonde have reported neural tube defects after exposure to high glucose. The strength of whole embryo culture is the ease with which it can be set up and observed. However, its use is restricted because it cannot address problems related to adult neuropathies.

### Embryonic stem cell line

Another system that deserves attention as a possible candidate for studies on diabetic neuropathy is the human embryonic stem cell line. Their unique property of pluripotency[68] allows the derivation of various neuronal cell types *in vitro*, directly or indirectly, and thus helps in the investigation of the molecular events underlying the toxic effects of glucose on various neuronal cell types. Human embryonic stem cells-derived embryoid bodies may also prove useful to address investigations regarding hyperglycemia-induced embryonic neuropathies, as they very closely mimic the early developing embryo.[69] Being derived from human embryos, these may prove to be immensely reliable and may resemble human tissue very closely. The main limitation of this model is the painstaking and delicate process of growing them on feeder layers[69,70] which increases the risk of intercellular contamination. Culture of stem cells on feeder free media containing high amount of human basic fibroblastic growth factor has been tried out.[68]

## Conclusion

A variety of model systems, each with its own set of advantages and limitations, are available for the study of diabetic neuropathy. *In vitro* models are useful to study intricate interaction of various molecules in the pathogenesis of diabetic neuropathy. An *in vivo* study would be required to confirm the occurrence of similar conditions in the presence of other interfering factors. Choosing an appropriate model system best suited for the study undertaken can reveal the pathogenesis of diabetic neuropathy and embryopathy and drugs targeting molecular events at different levels of the complication can be designed.
